# New changes in the journal

**DOI:** 10.3402/fnr.v60.34018

**Published:** 2016-11-30

**Authors:** Asim K. Duttaroy

Having completed my second year as Editor-in-Chief of *Food & Nutrition Research* (FNR), I wish to recognize the energy and passion of everyone involved in contributing to and operating this journal. Human capital is responsible for the creation of FNR as the dynamic publication of it spans from the authors who contribute their work to our editors to our reviewers – all of whom seek to work in a timely manner, while facing their own demanding time schedules. FNR is certainly ahead of the times, delivering up-to-date nutrition research that now reaches nearly the entire globe online. There are many great and powerful moments that go on behind the scenes of FNR and I have had the opportunity to both experience these moments and celebrate them over the last 2 years.

As 2016 comes to an end, we can conclude that it marks a successful and busy year. In terms of output, FNR has been productive, 100 papers published for the first time, which represents a significant growth in comparison with 2015.

In other areas of growth and change, our publisher Co-Action Publishing joined the Taylor & Francis Group, one of the world's leading publishing houses. I believe that this new publishing environment along with the continued support of the Swedish Nutrition Foundation will contribute actively to the further development and direction of FNR. As part of the many steps being taken to manage the growth of the journal, new editors are being appointed and the FNR editorial board is being updated. Editors and the board are expected to contribute actively to developing the journal and to its governance. We will be holding a virtual meeting in the coming months to disseminate all our plans and receive feedback from the board members.

On a personal level, I would like to acknowledge the considerable contribution and efforts of the editorial team, namely the editors Per Ole Iversen, Wendy Russell, and Kenneth Maleta and our newly appointed Statistical Advisor Abhik Ghosh. Moreover, I wish to express my gratitude to Veronica Svärd and Caroline Sutton of Co-Action Publishing, and Anneli Hovstadius and Nina Jansson from the Swedish Nutrition Foundation, for their considerable support and patience as we have grown the journal together. Looking forward, the focus of FNR will remain unchanged, namely on publishing Nutrition Research as outlined on the FNR website.

In conclusion, we thank all the authors and reviewers for their efforts in developing these studies to a satisfactory outcome. We invite nutrition researchers to submit their original work to FNR and to future special issues. Please do not hesitate to contact me or other members of the editorial team if you feel you can contribute to the further development of the journal.

Best wishes for a wonderful holiday season and have a very happy new year 2017!

*Asim K. Duttaroy*
Editor-in-Chief

